# Ozone

**Published:** 1868-03

**Authors:** 


OZONE.—It is now confidently asserted that, through the persevering labors of several distinguished chemists, the true nature of this important agent has been discovered. In the *Medical Times and Gazette*, Oct. 5, 1867, will be found a very interesting paper on this subject with a resumé of the various hypotheses on the nature of ozone, which have been propounded from the time of its first observer, Van Marum, in 1785, or of its real discoverer, Professor Schönbein, in 1840, down to the satisfactory solution of the present day.

Andrews and Tait, in a paper presented to the Royal Society in 1860, confirmed the previously known fact that only a small proportion, in extreme cases only one-twelfth, of the oxygen can by the electric discharge be converted into ozone, found that a constant and considerable diminution of volume accompanied the change. 100 volumes of oxygen, when subjected to the silent discharge, may contract to about 92 volumes. Hence ozone must be denser than oxygen. But another important fact was observed. Mercury, or some other oxidizable substance, was introduced into the ozonized oxygen, and the ozone was entirely absorbed. Strange to say, the oxygen which remained behind was found to have precisely the same volume as it had before the removal of the ozone. If 92 volumes of ozonized oxygen were so treated, 92 volumes of oxygen free from ozone would in all cases remain behind, so that the density of ozone appeared to be absolutely infinite. On the other hand, if the ozonized oxygen were heated, the original 100 volumes would be obtained, because, as every one knows, ozone is destroyed by heat.

Andrews and Tait did not attempt to account for this extraordinary fact; but soon afterwards Dr. Odling suggested an explanation which has recently been confirmed in a most striking manner by an experiment of Soret's. (*Comptes Rendus*, Nov. 27, 1865.) It is conceded by nearly all chemists that each molecule of oxygen in the free state consists of two atoms—that, in fact, the true formula for free oxygen is $O_2$. Odling suggested that the formation of ozone might really be the condensation of another atom of oxygen into each molecule, and that the formula for ozone might therefore be $O_3$, and its density one-half greater than that of oxygen. When 100 volumes of oxygen were reduced by ozonization to 92 it might be supposed that 8 volumes of oxygen combined with 16 volumes, and produced 16 volumes of ozone. The change might be represented in this way:

$$4O_2 + 8O_2 = 8O_3.$$

a molecule of ozone $O_3$ occupying the same volume as a molecule of oxygen $O_2$. The absorption of the ozone by mercury, iodine, etc., might really be only the removal of the third atom of oxygen, which would of course leave the volume unaltered.

$$\underset{\text{16 volumes}}{8O_3} + 8Hg = \underset{\text{16 volumes}}{8O_2} + 8HgO.$$

The same view would account for the mutual reduction which ozone and peroxide of hydrogen exercised upon one another, and, in fact, for all known re-actions of ozone.

$$\underset{\text{Ozone.}}{O_3} + \underset{\text{Peroxide of hydrogen.}}{H_2O_2} = \underset{\text{Oxygen.}}{2O_2} + \underset{\text{Water.}}{H_2O}.$$

This beautiful hypothesis, however, must have remained a mere hypothesis but for the remarkable experimental verification which it has received from the hands of M. Soret. We have seen that all ordinary substances are only capable of removing one atom of oxygen from each molecule of ozone; but Soret has at length succeeded in finding a body—oil of turpentine—which absorbs the whole molecule, the whole three atoms of oxygen. To take our previous illustration, if the 92 volumes of ozonized oxygen were treated with oil of turpentine, a dense white cloud would appear, the ozone would disappear, but instead of the volume remaining the same, it would contract to 76 volumes, the $16O_3$ having been removed bodily instead of being merely reduced to $16O_2$.

This experiment seems to place the matter beyond a doubt, and instead of the mass of hypotheses which so lately reigned, we have now a simple, beautiful, and coherent theory which affords an intelligible explanation of known facts. It is the more to be rejoiced at, since the importance of ozone in art as well as nature seems to be rapidly developing, and it is impossible to say how high that importance may rise.—*Medical and Library News.*

